# M344 Suppresses Histone Deacetylase-Associated Phenotypes and Tumor Growth in Neuroblastoma

**DOI:** 10.3390/ijms26178494

**Published:** 2025-09-01

**Authors:** Gabrielle L. Brumfield, Kenadie R. Doty, Shelby M. Knoche, Alaina C. Larson, Benjamin D. Gephart, Don W. Coulter, Joyce C. Solheim

**Affiliations:** 1 Eppley Institute, University of Nebraska Medical Center, Omaha, NE 68198, USA; gabrielle.brumfield@unmc.edu (G.L.B.); kedoty@unmc.edu (K.R.D.); shelby.knoche@sri.com (S.M.K.); alaina.larson@sanofi.com (A.C.L.); bgephart@unmc.edu (B.D.G.); 2 Fred & Pamela Buffett Cancer Center, University of Nebraska Medical Center, Omaha, NE 68198, USA; 3 Department of Pediatrics, University of Nebraska Medical Center, Omaha, NE 68198, USA; dwcoulter@unmc.edu; 4 Children’s Nebraska, Omaha, NE 68114, USA

**Keywords:** cancer, chemotherapy, histone deacetylase inhibitor, M344, neuroblastoma, pediatric cancer, preclinical

## Abstract

Neuroblastoma (NB) is an aggressive pediatric cancer, with high-risk patients facing a five-year survival rate of ~50%. Standard therapies, including surgery, chemotherapy, radiation, and immunotherapy, are associated with significant long-term toxicities and frequent relapse. Histone deacetylase (HDAC) inhibitors have emerged as promising agents for cancer therapy, given their role in modulating gene expression and tumor phenotypes. This study evaluated M344 [4-(dimethylamino)-N-(7-(hydroxyamino)-7-oxoheptyl)benzamide], an HDAC inhibitor, for its efficacy and mechanisms of action against NB. Analysis of clinical NB Gene Expression Omnibus data revealed advanced-stage tumors exhibit higher HDAC expression relative to early-stage samples. M344 treatment effectively increased histone acetylation, induced G0/G1 cell cycle arrest, and activated caspase-mediated cell death. Relative to vorinostat, an HDAC inhibitor in clinical use for lymphoma and clinical trials for NB, M344 displayed superior cytostatic, cytotoxic, and migration-inhibitory effects. In vivo, metronomic M344 dosing suppressed tumor growth and extended survival. Combination therapy with M344 and topotecan improved topotecan tolerability, while M344 co-administration with cyclophosphamide reduced tumor rebound post-therapy. In total, M344 demonstrated strong therapeutic potential for NB, offering improved tumor suppression, reduced off-target toxicities, and enhanced control of tumor growth post-therapy. These findings support further investigation of HDAC inhibitors, such as M344, for clinical application in NB treatment.

## 1. Introduction

Neuroblastoma (NB), a pediatric cancer derived from immature sympathetic nervous tissue, accounts for 15% of all pediatric cancer-related deaths [1]. With a diagnosis of NB, patients are stratified into one of three Children’s Oncology Group (COG) risk categories (low, intermediate, or high risk) based upon patient age at diagnosis, *MYCN* amplification status, histological features, tumor ploidy, clinical symptomology, extent of surgical resection, and International NB Risk Group Staging System (INRGSS) tumor stage [2]. Treatment for NB is largely determined by COG risk group, and regimens for patients may include surgical resection, high-dose chemotherapy (topotecan, cyclophosphamide, cisplatin, etoposide, vincristine, and doxorubicin), radiation therapy, stem cell transplantation, and immunotherapy [3]. Each component of treatment, while potentially lifesaving, carries risks that must be carefully managed. Survivors of NB may experience thyroid dysfunction, infertility, hearing loss, renal dysfunction, and/or secondary malignancy as a result of chemotherapeutic agents used during their clinical course [4]. Radiation exposure as a component of treatment puts NB patients at added risk for secondary malignancies and treatment-related sequelae [4]. Given that the average patient diagnosed with NB is under two years of age, adverse effects of treatment can have significant and life-long alterations to a patient’s development and wellbeing [5]. Beyond complications from therapy, the majority of NB patients achieve remission, but more than half of these patients will experience disease recurrence [6]. In part, relapse may be due to failure to remove residual disease and an inadequate immune response to the NB tumor. Pursuit of new therapeutic agents with a focus on maintaining disease-free status and reducing the toxicity of current regimens will not only increase the lifespan of NB patients but also their quality of life.

Histone deacetylase (HDAC) inhibitors are a class of agents that target HDAC enzymes, which remove acetyl groups from histones and thereby cause chromatin to condense into a conformation unfavorable for transcription. The 18 HDACs expressed by humans are divided by sequence homology into four subclasses: class I (HDAC1, 2, 3, 8), class II (HDAC4, 5, 6, 7, 9, 10), class III (sirtuin [SIRT]1, 2, 3, 4, 5, 6, 7), and class IV (HDAC 11) [7]. In the setting of cancer, increased HDAC activity leading to histone hypoacetylation may inhibit the expression of genes normally regulating cell cycle arrest, apoptosis, cellular motility, and immune regulation [8,9,10,11,12]. Altered expression of the class I and II HDAC enzymes is observed in solid tumors including NB, medulloblastoma, lung adenocarcinoma, renal, bladder, breast, and various cancers of the gastrointestinal and reproductive tracts [13]. Overexpression of HDAC enzymes in these cancers is largely associated with malignant transformation, advanced stage, and poor survival. It should be noted that select HDACs (1, 5, 6, 10) have an inverse relationship with low expression, correlating with poor prognosis in lung, gastric, cervical, and estrogen-receptor-positive invasive ductal breast cancers [13]. Specific to NB, upregulation of HDAC1, 2, 3, 5, 6, 8, and 10 is observed and associated with apoptosis suppression, tumor proliferation, drug resistance, increased metastasis, and decreased patient survival [12,14,15,16,17,18,19,20,21].

Inhibition of HDAC activity in NB has shown promise in preclinical studies to reverse these HDAC-associated phenotypes [12,14,15,16,18,20,21,22]. However, clinical studies utilizing HDAC inhibitors in NB are limited. Vorinostat, approved in 2006 by the U.S. Food and Drug Administration (FDA) for the treatment of cutaneous T-cell lymphoma, has been incorporated as a component of therapy for NB in clinical trials, along with radiotherapy, differentiating agents, and proteasome inhibitors, with varied responses [23,24,25]. The HDAC inhibitor M344 [4-(dimethylamino)-N-(7-(hydroxyamino)-7-oxoheptyl)benzamide] was shown by our lab to act as a multi-faceted therapy for the treatment of a pancreatic cancer mouse model, and treatment with M344 decreased the proliferation and viability of several neural-derived pediatric cancer cell types in studies by others [26,27,28]. No adverse effects were observed in mice treated with 10 mg/kg M344 daily for 4 months in a non-tumor-bearing mouse model, nor in our study of M344 treatment of a mouse pancreatic tumor model, thus supporting the safety of M344 use [27,29]. Additionally, in an HDAC inhibition assay, M344 was found to have IC_50_ values in the sub-micromolar range for HDAC1, 2, 3, 6, and 10, all of which are commonly overexpressed in NB [29]. M344 and vorinostat share a similar pool of class I and II HDAC targets with comparable IC_50_ potency against HDAC2, 3, and 10 in HEK293 cells, though M344 IC_50_ concentrations for HDAC1 and HDAC6 were, respectively, only 57% (0.048 μM versus 0.083 μM) and 35% (0.0095 μM versus 0.027 μΜ) of that required by vorinostat [29]. As of yet, the HDAC inhibitor M344 has not been reported as having been evaluated in preclinical in vivo studies for NB treatment.

With the knowledge that aberrant HDAC activity drives undesirable phenotypes associated with poor outcomes in NB, we evaluated the chemotherapeutic mechanisms of M344 as an HDAC inhibitor for reversal of HDAC-mediated characteristics in NB. Additionally, the efficacy and tolerability of M344 as a solo and combination therapy were investigated in vivo. Our preclinical characterization and assessment of M344 as a therapeutic for NB supports further exploration of this therapy to address the current high refractory rate and morbidity associated with this disease.

## 2. Results

**Figure 1:**
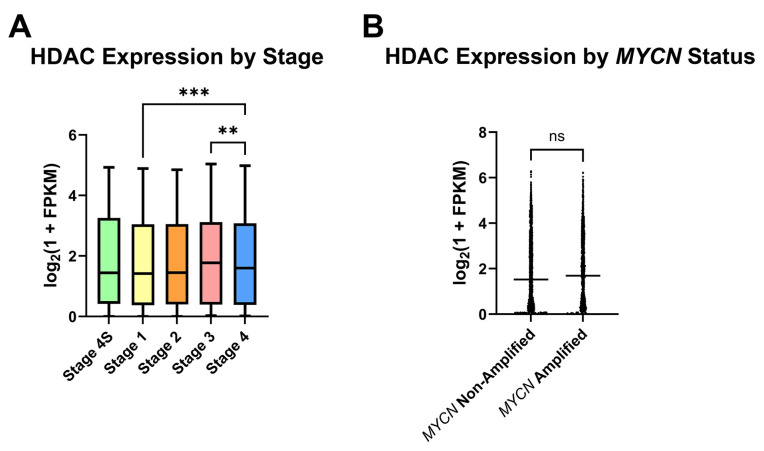
HDAC RNA transcripts are elevated in advanced-stage NB. High throughput RNA-sequencing data profiles of 498 primary NB samples (GEO accession series GSE49711) were assessed for HDAC expression (HDAC 1–11, SIRT 1–7) by log_2_(1 + Fragments Per Kilobase of transcript per Million mapped reads [FPKM]). (**A**) Clinical samples were pooled by INSS clinical stage (4S, 1, 2, 3, or 4) and analyzed for HDAC expression between stages. Statistical significance between groups was determined by one-way ANOVA with Tukey’s multiple comparisons test. (**B**) INSS stage 3 and 4 samples were stratified by *MYCN* status. Three samples (one at stage 3 and two at stage 4) were excluded from analysis due to no report of *MYCN* status. Statistical significance between groups was determined by unpaired *t*-test. ** *p* < 0.0, *** *p* < 0.001, ns = not significant.

For analysis of HDAC expression in human NB, a collection of Gene Expression Omnibus (GEO) clinical NB samples containing RNA-sequencing data from 498 primary tumors was appraised for HDAC expression in subgroups of NB, in part to assess which NB patient subpopulations may most benefit from HDAC inhibitor therapy. After stratifying samples according to the International Neuroblastoma Staging System (INSS) stages 1, 2, 3, 4, or 4S, detection of global HDAC transcripts was found to be largely conserved as the disease state progresses, and stage 4 samples had higher mean HDAC transcripts detected relative to stage 1 (Figure 1A). Due to the role that *MYCN* amplification plays in NB risk group placement and therefore patient outcomes, stage 3 and 4 samples were next segregated by *MYCN* status. No significant difference in HDAC transcripts was detected between *MYCN*-amplified and non-amplified samples (Figure 1B).

To initiate characterization of M344’s effects on preclinical NB, cell lines were treated within a range of concentrations found achievable in the plasma of in vivo models from previous studies. Mice treated with 10 mg/kg M344 achieved a peak plasma concentration of approximately 2700 ng/mL (~8.8 μΜ) [29]. Plasma concentration in mice treated with 10 mg/kg vorinostat is comparable, though slightly lower, at approximately 2000 ng/mL (~7.6 μΜ) [30]. Due to their similar rate of accumulation and clearance in mouse models and the lack of data concerning M344 pharmacokinetics and pharmacodynamics in humans, vorinostat human clinical trial data were considered to estimate M344 concentration in humans. Clinical studies in pediatric cancer patients treated with 230 mg/m^2^ vorinostat measured plasma concentrations from 461 ng/mL (~1.7 μΜ) to 2360 ng/mL (~8.9 μΜ) with a median of 780 ng/mL (~3.0 μΜ) [31]. Therefore, treatment with M344 was limited to 0–5 μΜ for the majority of our in vitro studies.

**Figure 2:**
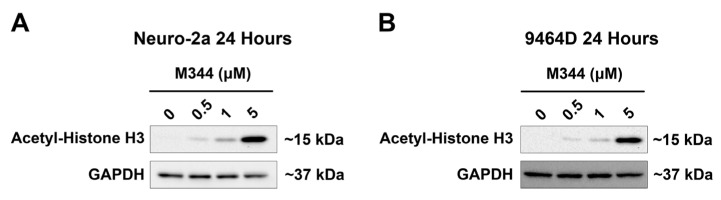
M344 treatment increases histone acetylation in NB cell lines. NB cell lines (**A**) lacking *MYCN* amplification (Neuro-2a) or (**B**) with *MYCN* amplification (9464D) were treated with 0.5–5 μΜ M344 or vehicle control (DMSO) for 24 h prior to whole cell lysate harvest and immunoblotting for acetyl-H3 and loading control, GAPDH.

For validation of M344-mediated inhibition of HDAC enzymes in NB cells, NB cell lines lacking *MYCN* amplification (Figure 2A, Neuro-2a) or harboring *MYCN* amplification (Figure 2B, 9464D) were treated for 24 h prior to immunoblotting for detection of acetylated histone H3. M344 treatment induced a steady increase in the acetylation status of histone H3 that correlated to M344 dosage, substantiating M344’s inhibition of HDAC enzymes in NB cells (Figure 2).

**Figure 3:**
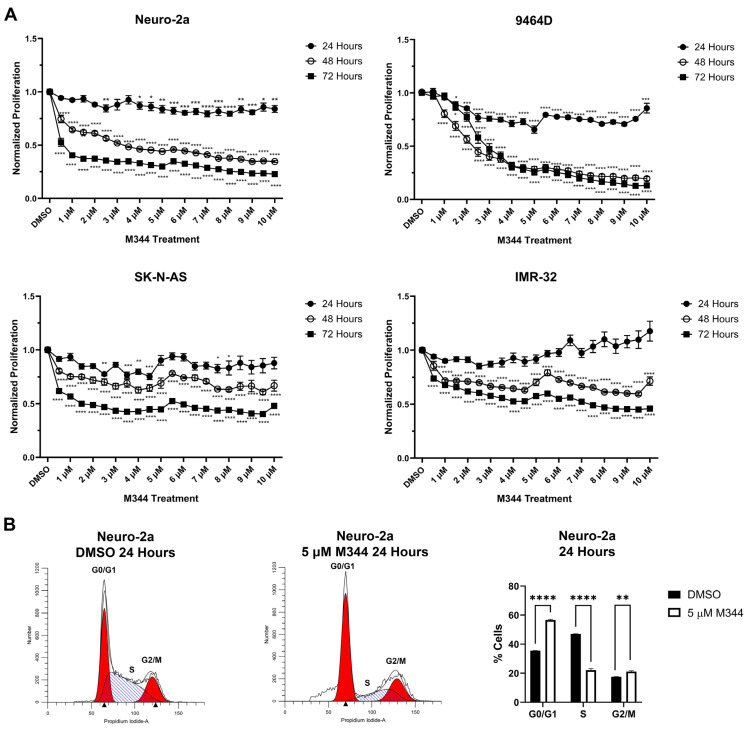
M344 inhibits NB cell proliferation through G0/G1 cell cycle arrest. (**A**) NB cell lines were treated with vehicle control (DMSO) or 0.5–10 μΜ M344 for 24, 48, or 72 h periods. Proliferation was monitored by MTT, and results are represented by mean ± standard error of the mean (SEM) (n = 3 per cell line and treatment concentration). Statistical significance was determined by one-way ANOVA with Dunnett’s multiple comparisons test. (**B**) Cell cycle analysis was performed by propidium iodide staining on Neuro-2a cells with 5 µM M344 for 24 h. Graphical presentation of flow cytometry results represented by mean ± SEM (n = 3). Statistical comparisons between DMSO control and 5 μΜ Μ344 groups were made by unpaired *t*-test. * *p* < 0.05, ** *p* < 0.01, *** *p* < 0.001, **** *p* < 0.0001.

**Figure 4:**
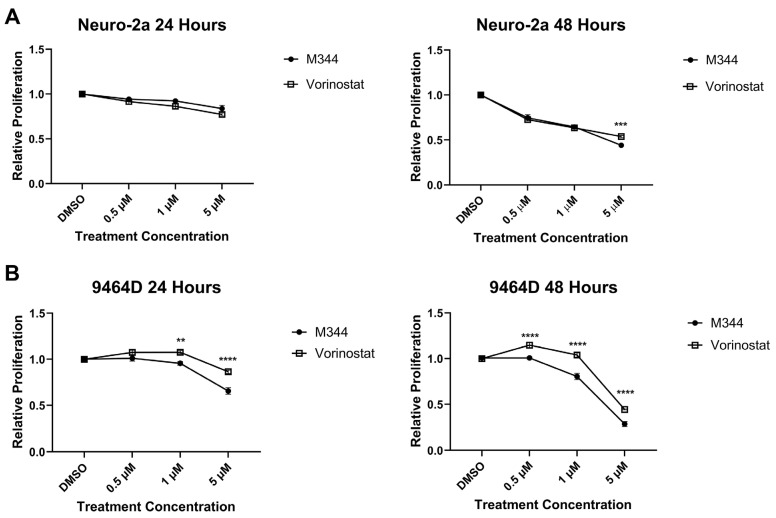
M344 outperforms vorinostat in inhibition of NB cell proliferation. (**A**) Neuro-2a and (**B**) 9464D NB cell lines were treated with vehicle control (DMSO) or 0.5–5 μΜ M344 or vorinostat for 24 or 48 h timepoints. Proliferation was monitored by MTT and results are represented by mean ± SEM (n = 3 per cell line and treatment concentration). Statistical significance between treatment groups was determined by unpaired *t*-test. ** *p* < 0.01, *** *p* < 0.001, **** *p* < 0.0001.

Evaluation of M344’s effects on the proliferation of NB cell lines by treatment with 4-(dimethylamino)-N-(1-(hydroxyamino)-7-oxoheptyl)benzamide (MTT) reagent in mouse and human *MYCN*-amplified (9464D and IMR-32) and non-amplified (Neuro-2a and SK-N-AS) cells lines revealed a stepwise decrease in NB cell proliferation in a time- and dose-dependent manner, affecting cell lines regardless of *MYCN* status (Figure 3A). Investigation of the mechanism of cytostatic activity through cell cycle analysis revealed a stark decrease in M344-treated cells in the S phase. A small number of cells accumulated in the G2/M phase, but a larger proportion of cells in the M344-treated group were found in G0/G1 (Figure 3B). The aggregation of cells in G0/G1, paired with the depletion of cells in the S phase, following M344 treatment suggests that M344 inhibits progression of NB cells from G0/G1 into the S phase, thereby preventing their division and further proliferation. In comparison of M344 to vorinostat for inhibition of NB cell proliferation, M344 outperformed vorinostat at doses as low as 0.5 μΜ, and a greater degree of benefit was seen for *MYCN*-amplified 9464D mouse NB cells (Figure 4B) versus *MYCN*-non-amplified Neuro2a mouse NB cells (Figure 4A).

**Figure 5:**
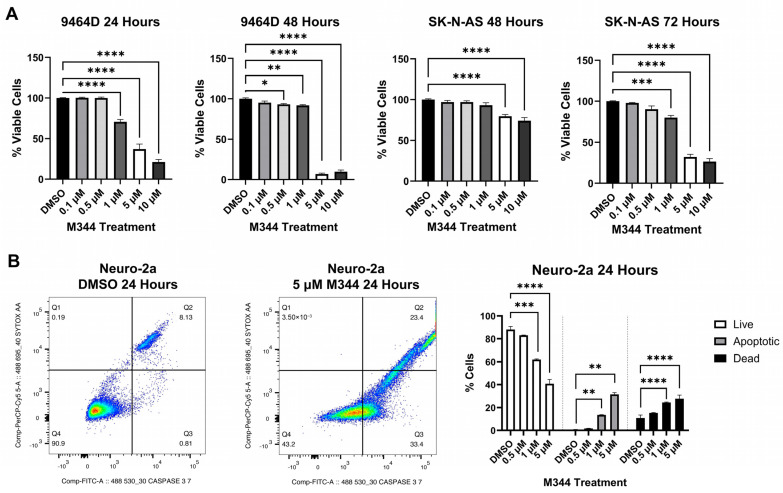
M344 diminishes the viability of NB cells through caspase-dependent mechanisms. (**A**) NB cell lines were treated for up to 72 h with DMSO vehicle, M344, or vorinostat at the indicated concentrations prior to trypan blue staining and counting via hemocytometer. Results are represented by mean [(viable cells/total cells) × 100] ± SEM (n = 4 per cell line and treatment concentration), and statistical significance of treatment relative to DMSO control was determined by one-way ANOVA and Dunnett’s multiple comparisons test. (**B**) Neuro-2a cells were treated with DMSO control or 5 μM M344 for 24 h. Caspase-3/7 cleavage (CellEvent Caspase-3/7 Green) and cell viability (SYTOX AADvanced Dead Cell Stain) were simultaneously analyzed by flow cytometry (n = 3). Density plots are color-coded with blue (low event density), green (intermediate event density), and red (high event density). Statistical significance of the results was determined by one-way ANOVA with Dunnett’s multiple comparisons test. * *p* < 0.05, ** *p* < 0.01, *** *p* < 0.001, **** *p* < 0.0001.

**Figure 6:**
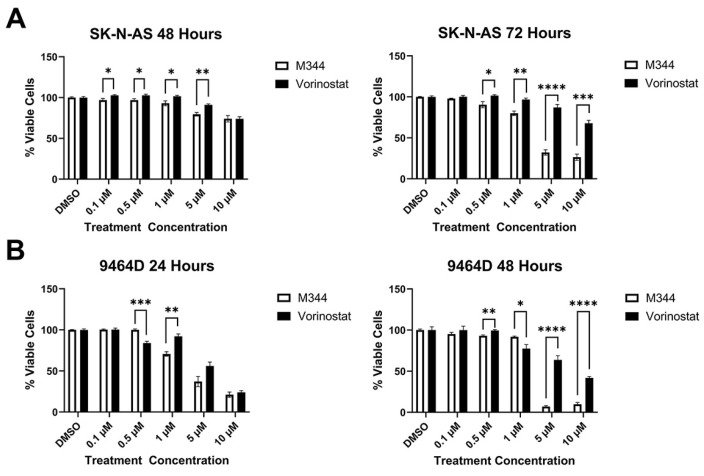
M344 is superior to vorinostat in reducing NB cell viability. NB cell lines were treated with vehicle control (DMSO), 0.1–10 μΜ M344, or 0.1–10 μΜ vorinostat for (**A**) 48 and 72 h for SK-N-AS or (**B**) 24 and 48 h for 9464D. Viability was determined by trypan blue staining and counting via hemocytometer. Results are represented by mean [(viable cells/total cells) × 100] ± SEM (n = 4–5 per cell line and treatment concentration), and statistical significance was determined by unpaired *t*-test. * *p* < 0.05, ** *p* < 0.01, *** *p* < 0.001, **** *p* < 0.0001.

Cytotoxicity of M344 to NB cells was assessed with trypan blue staining, and a reduction of viability by 50% or greater was achieved for both cell lines tested (mouse 9464D and human SK-N-AS cells) at 5 μΜ and 10 μΜ, respectively (Figure 5A). Subsequently, in flow cytometric staining with a nucleic acid viability dye (positive staining indicates dead cells) and activated caspase 3/7 (positive stain indicates active or completed apoptosis), an increasing proportion of cells relative to M344 treatment concentration stained single positive for activated caspase 3/7 (apoptotic) and double positive for activated caspase 3/7 and the nucleic acid viability stain (dead cells, apoptosis completed) (Figure 5B). Less than 0.5% of cells in all treatment groups stained single positive for nucleic acid viability stain, indicating non-apoptotic cell death. Thus, these results support caspase-mediated apoptosis as the cytotoxic mechanism for M344 killing of NB cells. M344 treatment produced a greater reduction in NB cell viability compared to vorinostat treatment for both SK-N-AS (Figure 6A) and 9464D cell lines (Figure 6B). At 72 h of treatment, vorinostat failed to reach the IC_50_ reached by M344 in SK-N-AS cells (Figure 6A).

**Figure 7:**
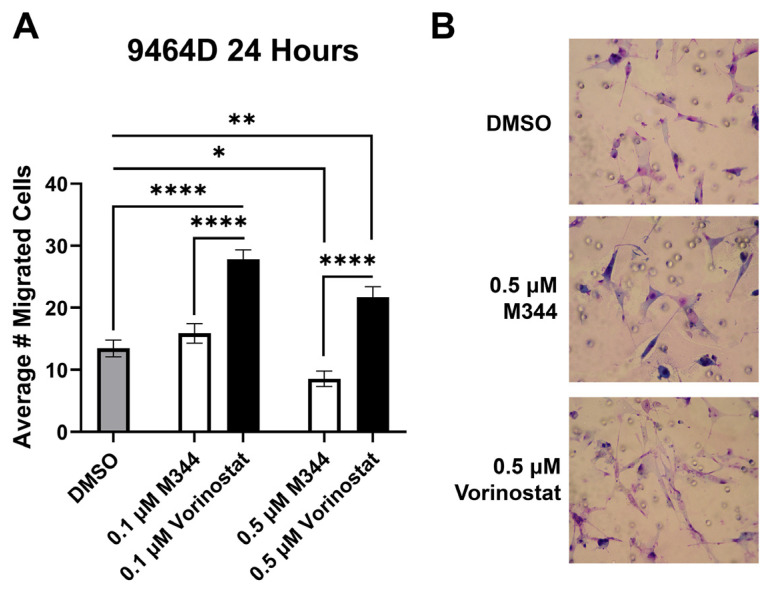
M344 inhibits migration of NB cells relative to vorinostat and vehicle control. 9464D cells were placed in 8-micron Transwell inserts with treatment, DMSO or select HDAC inhibitor, and allowed to migrate for 24 h. Migrated cells were stained, and images of stained inserts were collected with a Nikon Eclipse E400 microscope (Nikon Instruments, Melville, NY, USA) at 200× magnification for quantification. (**A**) Graphical results represent mean migrated cells ± SEM (DMSO n = 3, M344 n = 3 per concentration, vorinostat n = 2 per concentration). (**B**) Representative images from treatment groups. Statistical significance was determined by one-way ANOVA with Tukey’s multiple comparisons test. * *p* < 0.05, ** *p* < 0.01, **** *p* < 0.0001.

Nearly half of NB patients develop metastases, making inhibition of metastatic dissemination a desirable component of therapy [32]. Cancer cell migration contributes to metastatic capacity, and hence Transwell migration assays were utilized for assessment of NB cell migration in response to HDAC inhibitor treatment. Cells were treated with M344 or vorinostat at a maximum of 0.5 μΜ, a concentration found to not significantly reduce the proliferation or viability of the 9464D cell line after 24 h of exposure (Figure 3A and Figure 5A). After allowing cells to migrate for 24 h in media containing their respective HDAC treatment, 0.1 μΜ M344 did not significantly reduce 9464D migration, but an increase in migration was observed in the vorinostat-treated cells (Figure 7). Exposure to 0.5 μΜ vorinostat enhanced cell migration relative to vehicle control, though at this concentration some reduction in migration was seen for M344-treated cells compared to the dimethyl sulfoxide (DMSO) control (Figure 7). At both concentrations, the average number of migrated cells was lower in the M344 group than in the vorinostat-treated group (Figure 7).

**Figure 8:**
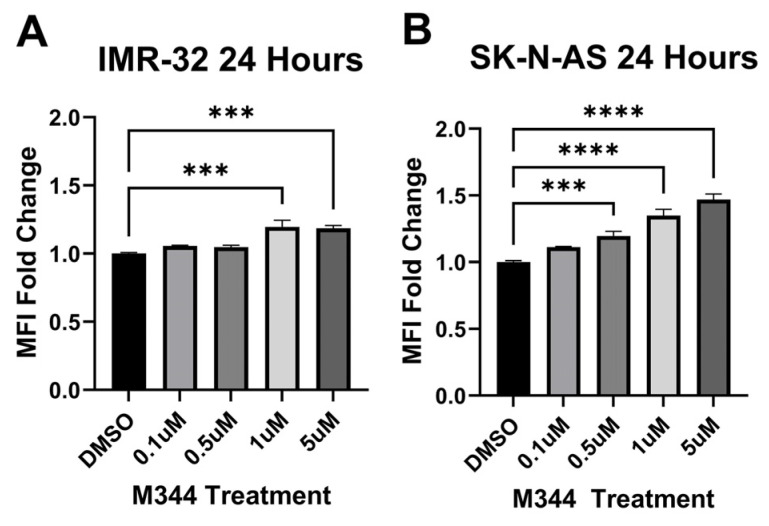
M344 enhances surface MHC I expression on NB cells. NB cells (**A**) with *MYCN* amplification, IMR-32, or (**B**) lacking *MYCN* amplification, SK-N-AS, were treated with 0.1–5 μΜ M344 or vehicle control (DMSO) and analyzed by flow cytometry for surface MHC I expression. Graphical results represent median fluorescence intensity (MFI) ± SEM (IMR-32 n = 2, SK-N-AS n = 3). Statistical significance was determined by one-way ANOVA with Dunnett’s multiple comparisons test. *** *p* < 0.001, **** *p* < 0.0001.

**Figure 9:**
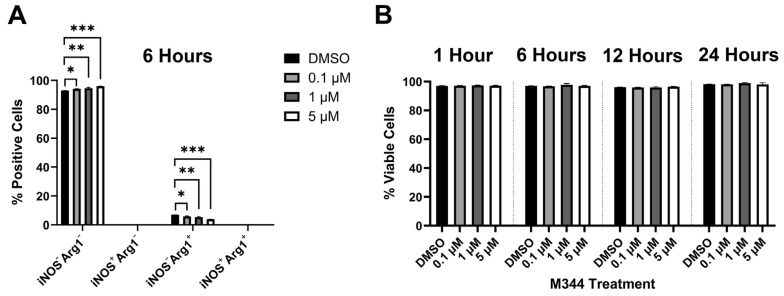
M344 treatment reduces M2/anti-inflammatory macrophage polarization without reduction in macrophage viability. Primary macrophages were collected from normal C57BL/6 mice by treatment of tibia and fibula bone marrow with M-CSF-containing L929-conditioned media for 7 days. C57BL/6 bone marrow-derived macrophages were treated with 0.1–5 μΜ Μ344 and mature macrophages (CD11b^+^F4/80^+^ cells) were evaluated by flow cytometry for (**A**) iNOS (pro-inflammatory)/Arg1 (anti-inflammatory) markers and for (**B**) viability with amine-reactive viability dye. Graphical results represent mean positive cells ± SEM (n = 3). Statistical significance was determined by one-way ANOVA with Dunnett’s multiple comparisons test. * *p* < 0.05, ** *p* < 0.01, *** *p* < 0.001.

Relapse and metastatic dissemination are both in part due to the failure of the immune system to recognize and eliminate NB cells. We assessed M344’s efficacy in relieving two immune-suppressive influences relevant to the NB tumor microenvironment: reduced surface presence of major histocompatibility complex class I (MHC I) molecules on NB cells (leading to decreased detection by T cells) and the presence of anti-inflammatory macrophages (which perpetuate NB tumor growth in part through suppression of ant-tumor immune cells) [33]. M344 treatment of human NB cell lines IMR-32 and SK-N-AS induced an increase in surface MHC I detected by flow cytometry (Figure 8). For our evaluation of inflammatory status of M344-treated macrophages, we utilized the pro- and anti-inflammatory markers inducible nitric oxide synthase (iNOS) and arginase-1 (Arg1), respectively. Among normal bone marrow-derived macrophages, a baseline population of cells expressing the M2/anti-inflammatory macrophage-associated marker Arg1 is expected [34]. Treatment of normal bone marrow-derived murine macrophages with 0.1–5 μΜ M344 reduced the population of M2/anti-inflammatory iNOS^−^Arg1^+^ cells and increased the proportion of unpolarized (M0) iNOS^−^Arg1^−^ macrophages (Figure 9A), without a reduction in viability of these primary immune cells (Figure 9B).

**Figure 10:**
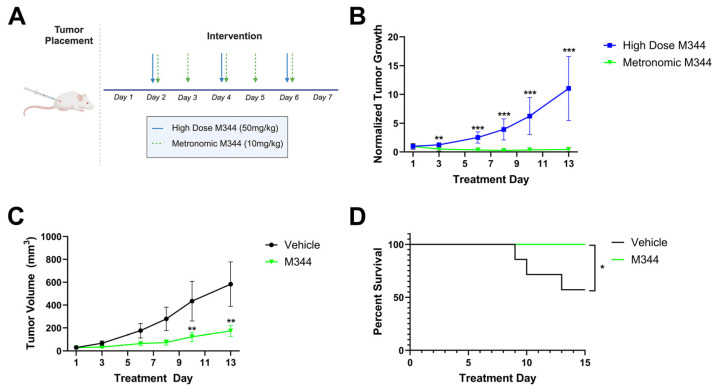
Metronomic M344 treatment reduces NB tumor growth and extends survival in vivo. (**A**) Treatment timeline for assessment of M344 in a subcutaneous NB mouse model. Following tumor inoculation and growth to trial threshold, mice were randomly assigned to treatment group. Mice received either high-dose M344 (50 mg/kg 3 times per week, n = 3) or metronomic M344 (10 mg/kg 5 times per week, n = 8) for 2 weeks (total of 11 mice treated in the experiment). (**B**) Tumor growth rates for mice treated with high-dose and metronomic M344 were calculated by normalization of group tumor volume at indicated treatment days to Day 1 tumor volume. Statistical significance was determined by unpaired t-test. (**C**) Metronomic M344 group tumor volume (n = 8) compared to mice treated with vehicle (DMSO equal to M344 stock volume with 5% Tween 80 in PBS, n = 7) (total of 15 mice treated in the experiment). Statistical significance was determined with mixed-effects analysis. (**D**) Kaplan–Meier survival analysis of M344- versus vehicle-treated mice. * *p* < 0.05, ** *p* < 0.01, *** *p* < 0.001.

Following in vitro assessment of chemo- and immunotherapeutic profiles and mechanisms of M344, we utilized an in vivo NB model to investigate M344 dosing regimens and efficacy in tumor growth suppression. A/J mice were injected subcutaneously with 1 × 10^6^ syngeneic Neuro-2a cells and monitored for tumor development. Following the detection of palpable tumors, mice were divided into treatment groups that either received high dose M344 (50 mg/kg intraperitoneally [IP] three times per week, n = 3) or a metronomic M344 regimen (10 mg/kg IP 5 times per week, n = 8) over a 13-day treatment period (Figure 10A). Comparing tumor volumes normalized to the Day 1 volume in each group, mice treated with metronomic dosing of M344 had greater tumor growth suppression relative to mice treated with high dose M344 starting at treatment Day 3 and continuing to Day 13 (Figure 10B). Relative to vehicle control (5% Tween 80 in PBS with DMSO equal to M344 stock volume, n = 7), metronomic M344 significantly reduced tumor growth (Figure 10C) and extended survival (Figure 10D). Thus, finding the higher dose and lower frequency treatment regimen to be inferior, we adopted the metronomic dosing scheme in our subsequent in vivo studies.

**Figure 11:**
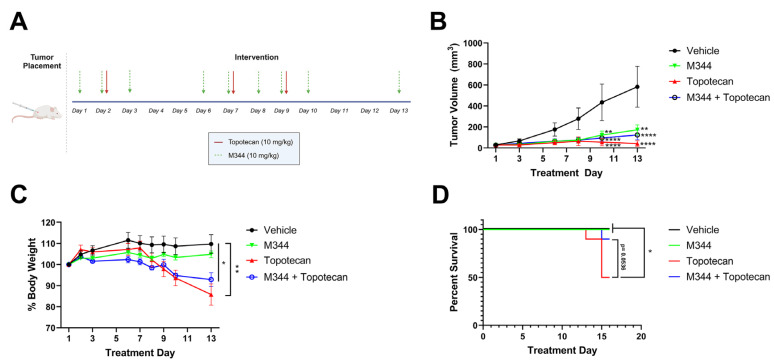
M344 increases tolerability of topotecan therapy. (**A**) Mice with subcutaneous NB tumors were treated with vehicle (DMSO, 5% Tween 80 in PBS, n = 10), topotecan (10 mg/kg, 3 doses over 9 days, n = 10), M344 (10 mg/kg 5 times per week, n = 11), or combination therapy with M344 + topotecan (n = 10) for 2 weeks (total of 41 mice treated in the experiment). (**B**) Graphical representation of mean group tumor volume ± SEM. Statistical significance was determined with mixed-effects analysis. (**C**) Body weight of mice was recorded 5 times per week during treatment period. Statistical significance was determined by mixed-effects analysis. (**D**) Kaplan–Meier survival analysis of vehicle, M344, topotecan, and combination therapy groups. * *p* < 0.05, ** *p* < 0.01, **** *p* < 0.0001.

After optimizing our dosing of M344, we moved to investigate the capability of M344 to address two major challenges in NB therapy development, i.e., the need to reduce off-target toxicities and sustain the therapeutic response. For tolerability studies, body weight loss exceeding 20% of starting weight or morbidity scoring of mice below acceptable thresholds, as determined by trained veterinary staff at the University of Nebraska Medical Center, were criteria for euthanasia. Topotecan, a topoisomerase I inhibitor used as a standard chemotherapy for the treatment of high-risk NB, was given to mice at 10 mg/kg IP in three doses over 9 days as a solo therapy (n = 10), or in combination with metronomic M344 delivered 5 days per week (n = 10). Additional mice were treated with M344 solo therapy (n = 11) or vehicle control (n = 10) (Figure 11A). A similar degree of tumor control was achieved in topotecan, M344, and combination therapy groups, with all groups achieving significantly reduced tumor volumes relative to vehicle control at Day 10 and 13. Additionally, there were no significant differences in the groups’ tumor volumes between the M344, topotecan, and combination therapy groups (Figure 11B). Mice treated with topotecan solo therapy experienced significant weight loss relative to vehicle control (Figure 11C), and beginning at Day 13, the weight loss of a subset of mice in the topotecan therapy group met euthanasia criteria by exceeding the threshold of greater than 20% loss of starting body weight. Overall, 5/10 mice in the topotecan solo therapy group experienced >20% body weight loss, whereas weight loss was attenuated in the combination therapy group with only 1/10 mice reaching the 20% body weight loss threshold (Figure 11C). Survival of topotecan-treated mice was significantly reduced in comparison to the vehicle and M344 solo therapy groups, and the combination therapy group trended toward extending survival relative to topotecan solo treatment (*p* = 0.0536) at the termination of the study (Figure 11D). These findings support metronomic M344 as a therapy with a more tolerable toxicity profile and suggest that concurrent delivery of M344 may have potential as a means of limiting topotecan toxicity in NB treatment.

**Figure 12:**
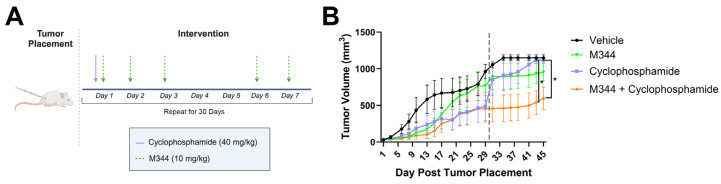
After completion of therapy, tumor rebound is suppressed in mice that received M344 and cyclophosphamide combination treatment relative to cyclophosphamide solo treatment. (**A**) Mice with subcutaneous NB tumors were treated with vehicle (DMSO, 5% Tween 80 in PBS, n = 7), cyclophosphamide (40 mg/kg 1 time per week, n = 8), M344 (10 mg/kg 5 times per week, n = 8), or combination therapy with M344 + cyclophosphamide (n = 10) for 30 days (total of 33 mice treated in the experiment). Tumor volumes were recorded for an additional 15 days after cessation of treatment. (**Β**) Graphical representation of mean group tumor volumes ± SEM. Vertical dashed line indicates the end of treatment. Statistical significance was determined with mixed-effects analysis. * *p* < 0.05.

Patients with advanced NB often have an initial robust response to therapy but frequently relapse [6]. With the goal of creating a durable response to therapy, we treated tumor-bearing mice with 40 mg/kg IP cyclophosphamide once per week (n = 8), M344 (n = 8), vehicle control (n = 7), or a combination of cyclophosphamide with M344 (n = 10) (Figure 12A). Mice were treated for 30 days, and after completion of therapy, the tumor-bearing mice were monitored for an additional 15 days for tumor growth. From Day 31 to 45, only the combination M344 and cyclophosphamide therapy group had a significant reduction in group tumor volume compared to the vehicle control, and on Day 43 and 45 the average tumor volume of the M344 and cyclophosphamide combination therapy group was significantly lower than the cyclophosphamide solo therapy group (Figure 12B). Thus, M344 may act as a useful adjunct to cyclophosphamide treatment of NB for sustained tumor control.

## 3. Discussion

Treatment planning for NB patients currently relies on information utilizing the disease stage in addition to clinical and histologic characteristics. In our investigation of the HDAC inhibitor M344 as a treatment modality for NB, we analyzed primary NB patient samples for HDAC expression to assess which NB patient populations may receive benefit from HDAC inhibitor therapy. Our analysis of HDAC transcript expression across INSS stages in primary NB samples demonstrated that HDAC transcripts were detected in all INSS stages of NB, with the highest mean HDAC transcripts in stage 3 and 4 patients (Figure 1A). After stratifying advanced-stage patients by *MYCN* status, we saw no statistical difference between *MYCN*-amplified and non-amplified groups (Figure 1B). These data suggest that increased global HDAC expression may occur independently of *MYCN*-mediated effects, and in vitro assessment of H3 histone acetylation validated that M344 treatment increases histone acetylation in both *MYCN*-amplified and non-amplified NB cell lines (Figure 2). Expression of HDAC8 has previously been positively correlated with NB stage, but our work provides further insight into global trends of HDAC expression in NB [15]. Consequently, while further studies are required to assess the clinical safety and efficacy of M344 before use in humans, our analysis of HDAC transcripts in NB samples suggest that in the treatment of NB, patients with advanced-stage tumors, regardless of *MYCN* status, may receive the most substantial benefit from HDAC inhibitor therapy.

Further in vitro investigation of M344’s cytotoxic and cytostatic effects displayed a significant decrease in NB cell proliferation and viability with treatment (Figure 3 and Figure 5). Mechanisms of cytotoxic activity (apoptotic cell death, Figure 5B) align with those reported for vorinostat in NB cells [35,36]. However, compared to cytostatic G0/G1 cell cycle arrest of M344-treated NB cells (Figure 3B), vorinostat-treated NB cell lines were found to arrest in G0/G1 or G2/M [35,36]. The increased anti-proliferative and pro-apoptotic effects observed in M344-treated NB cells (Figure 4 and Figure 6) may be mediated through enhanced suppression of HDAC1, a known mediator of cell cycle checkpoint progression and apoptosis, as M344 HDAC1 IC_50_ is roughly half of that required by vorinostat in HEK293 cells [9,29,37]. Generation of individual HDAC knockdowns in M344-treated NB cell lines would provide valuable insight in further delineation of the role of HDAC1 and other class I and II HDACs in M344’s cytotoxic efficacy.

We observed increased migration of vorinostat-treated NB cells (Figure 7), and a similar phenomenon has also been reported for cell lines derived from lung, liver, and breast cancers [38,39,40]. Other HDAC inhibitors have shown a reduction in migration and metastatic capacity of cell lines, and therefore the direction and degree of migration modulation may be dependent on cell type and pharmacologic profile of the HDAC inhibitor [41,42]. In our studies, M344 significantly reduced migration of NB cells at sub-cytostatic and -cytotoxic doses (Figure 3A, Figure 5B and Figure 7). However, at equivalent dosing, vorinostat enhanced Transwell migration (Figure 7). These divergent effects may be attributed to differing inhibition of HDAC6 and HDAC11 by M344 and vorinostat. While inhibition of HDAC6 may prevent cell migration, inhibition of HDAC11 has been shown to increase the migratory activity of cancer cells [40,43]. HDAC6 has been shown to play a role in cytoskeletal remodeling required for cellular migration, and these functions are impaired in HDAC6-deficient cells [43]. The mechanism by which inhibition of HDAC11 encourages cancer cell migration is less clear. However, inhibition of HDAC11 in animal models of breast cancer increases metastatic dissemination, hypothesized to be due to increased expression of the pro-migratory protein ribonucleotide reductase M2 in breast cancer cells [40]. M344 achieves IC_50_ inhibition of HDAC6 at a concentration roughly 70% lower than vorinostat in HEK293 [29]. Relatively, M344 may be a poor inhibitor of HDAC11 (18% inhibition at 100 μΜ in HEK293) in comparison to vorinostat, which significantly upregulated HDAC11-target genes in breast cancer cells at 0.5 μΜ [29,44]. Therefore, the paradoxical effects on migration displayed by M344 and vorinostat may be caused by M344’s greater degree of HDAC6 inhibition and lesser impact on HDAC11. Our data clarify the migratory effects of both M344 and vorinostat on NB cells and support further work in assessing the inhibitory profile of M344 as a candidate for clinical prevention of NB metastatic spread.

The success of efforts to generate and enhance immune responses against NB via immunotherapies may be reduced by suppressive immune cells, such as M2/anti-inflammatory macrophages, and by low surface MHC I, limiting the detection of tumor cells by T cells. Increased infiltration of M2 macrophages has been observed in advanced-stage NB, and the activity of HDACs may alter macrophage inflammatory status [45,46]. We found that with M344 treatment of primary macrophages, there was a reduction in the presence of M2/anti-inflammatory macrophages (iNOS^−^Arg1^+^), and these cells reverted to an unpolarized (iNOS^−^Arg^−^) status (Figure 9A) without a reduction in macrophage viability (Figure 9B). In the NB tumor microenvironment, this M344 activity may alleviate macrophage-mediated immune suppression, thereby enhancing the activity of tumor-reactive immune cells. Furthermore, M344 shifted macrophages to an unpolarized state, which may allow repolarization toward an anti-tumor phenotype. Work in autoimmune and inflammatory disorders reports that HDAC inhibition may suppress inflammatory responses at lower concentrations, but effects such as regulation of pro-inflammatory IL-8 release may diverge with cell type, treatment concentration, and the selected HDAC inhibitor [47,48,49,50]. Though we focused on relieving immunosuppressive factors in this study, further studies are needed to understand M344’s effects on pro-inflammatory subsets of macrophages and other immune cells. Additionally, we found that M344 treatment enhanced surface MHC I expression on NB cells (Figure 8), which would enable better recognition of these cancer cells by tumor-infiltrating T cells. This finding is congruent with the literature highlighting HDAC-mediated MHC I downregulation and reversal of this phenotype with HDAC inhibitor treatment [11,27,51]. Together, these findings suggest that there may be immunological benefits of M344 as a component of NB therapy. Functional studies demonstrating that elevated MHC I expression induced by M344 treatment mediates increased T cell-dependent tumor cell death would strengthen evidence for M344 as an immunotherapeutic agent. Our animal studies utilized a murine cell line in syngeneic, wild-type mice. Additional studies with human xenograft data, ideally in humanized mice, would provide further clarity and specificity to the potential immunotherapeutic mechanisms of M344 in treating human NB.

Initial evaluation of M344 dosing schemes in vivo (Figure 10A) indicated superior tumor growth suppression with metronomic M344 relative to high-dose M344 (Figure 10B) and vehicle control (Figure 10C), in addition to significant extension of survival relative to vehicle control (Figure 10D). However, here a relatively small sample size of mice received high-dose M344 (n = 3), and larger studies evaluating pharmacodynamic properties of M344 would be beneficial to understanding optimal M344 dosing. Current pharmacokinetic data from mouse studies suggest that M344 and vorinostat may have a relatively similar rate of elimination, and human studies estimate vorinostat’s half-life to be 2.4 h [29,30,31]. Magnitude of effect increased with exposure time to M344 in many of our in vitro studies (Figure 2, Figure 3A, Figure 4, Figure 5 and Figure 6). If M344’s half-life is presumed to be similar to that of vorinostat at 2.4 h in humans, the increased effect observed in five-times-per-week metronomic dosing may be due to increased exposure time of tumor cells to M344, despite the total dosing of M344 being lower. The enhanced efficacy of metronomic dosing likely reflects increased exposure time of tumor cells to M344, suggesting that exposure duration may be more critical than high-dose scheduling. In conjunction with our studies on NB cell lines, these data support the effects of M344 as being both time- and dose-dependent, and to produce maximal therapeutic effect in vivo, exposure time is prioritized over high dose in metronomic treatment. Reflective of previous studies [27,29], we successfully treated mice with M344 for up to 30 days without a significant reduction in body weight and/or signs of gross toxicity. However, additional studies are required to fully understand the impact of M344 on key organs.

With M344 dosing optimized, we moved to address the addition of M344 to NB therapy as a potential strategy for enhancing efficacy and reducing toxicities of standard chemotherapy treatments. In our studies, M344 increased the tolerability of topotecan therapy (Figure 11). Topotecan, a topoisomerase I inhibitor, acts in the S phase of the cell cycle to induce single-stranded breaks in DNA [52]. Inhibition of class I HDACs inhibits DNA-damage responses in multiple cancer types, though in normal fibroblasts inhibition of class I HDACs was protective against double-stranded breaks induced by topoisomerase II inhibitors [53,54,55]. These divergent effects have been connected to inhibition or activation of DNA repair within malignant and non-transformed cells. In non-transformed fibroblasts, class I HDAC inhibition enhances expression of genes related to DNA damage repair [55]. Due to defects in DNA repair that occur in the pathogenesis of cancerous transformation, activation of these same DNA-damage repair mechanisms is unavailable to transformed cells [53,55]. Inactivation of Mre11 in transformed fibroblasts was identified as a potential causative agent, and dysregulation of Mre11 has been identified in NB [54,55,56]. Therefore, M344 may be protective against topotecan-induced DNA damage in non-cancerous tissue, which in our studies prevented significant body weight loss necessitating euthanasia in mice (Figure 11C), without inhibiting topotecan-mediated DNA damage in NB tumors to suppress tumor growth (Figure 11B).

The addition of M344 treatment to a regimen of cyclophosphamide, an alkylating agent, also attenuated post-therapy tumor rebound compared to mice treated with solo therapy (Figure 12) [57]. Inhibition of HDAC1 sensitizes NB cells to DNA-damaging agents, and HDAC10 has been identified as a mediator of resistance to DNA-damaging chemotherapeutics in NB [17,21]. Paired with the potential DNA damage mechanism shared with topotecan and M344 dual therapy, targeting both HDACs with M344 therapy may improve cyclophosphamide-mediated DNA alkylation, resulting in restricted tumor growth. Additionally, low-dose cyclophosphamide has demonstrated the ability to deplete regulatory T cells in rodent models and clinical trials with metastatic breast and colorectal cancer patients, where an increase in effector T-cell subsets was also observed [58,59,60,61]. The shift toward tumor-reactive T cells mediated by cyclophosphamide with a complementary increase in surface MHC I expression on tumor cells and inhibition of anti-inflammatory macrophages due to M344 (Figure 8 and Figure 9A) may also aid in suppressing tumor rebound. Although we showed M344’s ability to diminish tumor rebound after cessation of therapy, future animal studies with chemotherapeutic regimens optimized to achieve complete regression will be needed to more fully assess M344’s role in maintaining disease-free status and preventing relapse of NB.

## 4. Materials and Methods

### 4.1. Reagents

M344 (APExBio, Houston, TX, USA catalog no. A4105 and Selleck Chemicals, Houston, TX, USA catalog no. S2779) and vorinostat (APExBio, catalog no. A4084) were solubilized in DMSO (Thermo Fisher Scientific, Waltham, MA, USA catalog no. BP231100). Topotecan (Selleck Chemicals, catalog no. S1231) and cyclophosphamide (Selleck Chemicals, catalog no. S2057) were dissolved in phosphate-buffered saline (PBS, Thermo Fisher Scientific, catalog no. 10-010-031). Vehicle controls for in vitro (DMSO) and in vivo (PBS + DMSO) use were prepared with equivalent DMSO volume to M344 or vorinostat stocks. Stocks were stored at −80 °C until use.

### 4.2. Cell Lines and Culture Conditions

NB cell lines derived from human (IMR-32 and SK-N-AS) and mouse (9464D and Neuro-2a) tumors were used in these studies. The 9464D mouse cell line is derived from the transgenic TH-MYCN mouse with *MYCN* amplification, and Neuro-2a cells are derived from a spontaneous murine NB tumor lacking *MYCN* amplification [62,63]. Human IMR-32 cells were isolated from an abdominal, *MYCN*-amplified NB mass in a 13-month-old male, and human SK-N-AS cells were developed from a metastatic, *MYCN*-non-amplified NB found in the bone marrow of a 6-year-old female [64]. All cell lines were regularly tested and confirmed to be mycoplasma-free using a mycoplasma detection kit (Applied Biological Materials, Richmond, BC, Canada catalog no. G238). Human NB cell lines were cultured in Life Technologies RPMI 1640 (Thermo Fisher Scientific, catalog no. 11875119), and mouse-derived NB cell lines were maintained in Life Technologies DMEM (Thermo Fisher Scientific, catalog no. 11965092). Media for all cell lines were supplemented with the following for generation of complete media: 10% fetal bovine serum (FBS) heat inactivated at 56 °C for 30 min (R&D Systems, Minneapolis, MN, USA catalog no. S11550), 10 mM HEPES (Thermo Fisher Scientific, catalog no. 15630-080), 2 mM L-glutamine (Thermo Fisher Scientific, catalog no. 25030-081), 1 mM sodium pyruvate (Thermo Fisher Scientific, catalog no. 11360070), 1 × non-essential amino acids (Thermo Fisher Scientific, catalog no. 1140050), 100 units/mL penicillin (Thermo Fisher Scientific, catalog no. 15140122), and 100 μg/mL streptomycin (Thermo Fisher Scientific, catalog no. 15140122).

### 4.3. RNA-Sequencing Data Analysis

High-throughput RNA-sequencing data profiles of 498 primary human NB samples GEO accession series GSE49711 were assessed for HDAC expression (HDAC 1–11, SIRT 1–7). Detected transcripts were pooled by INSS clinical stage (1, 2, 3, 4, or 4S). NB samples with clinical stage 3 and 4 were separately analyzed for HDAC expression by *MYCN* amplification status (non-amplified or amplified).

### 4.4. Immunoblotting

Cells collected for immunoblotting were removed from flasks with scraping and further lysed in RIPA buffer (Thermo Fisher Scientific, catalog no. 89901) supplemented with 2 mM dithiothreitol (MilliporeSigma, Burlington, MA, USA catalog no. 43816), 0.1 mM phenylmethylsulfonyl fluoride (MilliporeSigma, catalog no. 10837091001), 1 mM Na_3_VO_4_ (MilliporeSigma, catalog no. S6508), and 1 mg/mL Halt Protease Inhibitor Cocktail (Thermo Fisher Scientific, catalog no. 78430). Collected lysates were frozen overnight at −80 °C, thawed, and centrifuged at 13,000 RPM for 30 min at 4 °C using an Eppendorf 5417R centrifuge. Thawed samples were mixed with 5× dye (10% *w*/*v* sodium dodecyl sulfate [Bio-Rad Laboratories, Hercules, CA, USA catalog no. 1610302], 30% *v*/*v* glycerol [MilliporeSigma, catalog no. G2025], 250 mM Tris [Thermo Fisher Scientific, catalog no. BP15201], 0.2% *w*/*v* bromophenol blue [MilliporeSigma, catalog no. B-5525], and 5% *v*/*v* β-mercaptoethanol [MilliporeSigma, catalog no. M-7522]) before being boiled for 5 min at 95 °C. Samples were loaded onto Invitrogen Novex Tri-glycine polyacrylamide pre-cast gels (Thermo Fisher Scientific, catalog no. XP04205BOX) and electrophoresed at 100 volts for 2 h. Proteins were transferred to an Immobilon-P Millipore polyvinylidene difluoride membrane (MilliporeSigma, catalog no. IPVH00010) at 30 volts for 1 h and 50 min. To block non-specific binding, the membrane was incubated at room temperature in a 5% *w*/*v* nonfat dry milk solution. The primary antibodies (glyceraldehyde-3-phosphate dehydrogenase [GAPDH] [MilliporeSigma, catalog no. G8795] and acetyl-Histone H3 [Cell Signaling Technology, Danvers, MA, USA catalog no. 9677S]), were added to the blocking solution, and the membrane was incubated overnight at 4 °C. The following day, the membrane was washed 3 times with 0.1% Tween-20 (Thermo Fisher Scientific, catalog no. BP337-500) in Tris-buffered saline pH 7.4 (Thermo Fisher Scientific, catalog no. BP2471-1) for 5 min per wash at room temperature. An appropriate secondary antibody (Jackson ImmunoResearch, West Grove, PA, USA catalog no. 115-035-174 and 211-032-171) was then applied, and the membrane was incubated in the antibody solution for 1 h at room temperature, followed by 3 additional washes as described. The membrane was incubated for 3 min at room temperature in SuperSignal West Pico PLUS Chemiluminescent Substrate (Thermo Fisher Scientific, catalog no. 34580), and protein bands were visualized using the Bio-Rad ChemiDoc Imaging System B and Image Lab software version 6.1 (Bio-Rad Laboratories).

### 4.5. MTT Proliferation Assay

Proliferation of NB cells was assessed at 24, 48, and 72 h timepoints with 2500 cells/wells seeded in 96-well plates. Cells were allowed to attach for 24 h following initial plating, after which they were treated with indicated drug concentrations in 90 μL total volume with 5 technical replicates per treatment concentration. At each final timepoint, 30 μL of 2 mg/mL MTT reagent (thiazolyl blue tetrazolium bromide, Thermo Fisher Scientific, catalog no. L11939) was added to each well followed by a 3 h incubation at 37 °C. After removal of all liquid from the wells, 300 μL isopropanol (Avantor, Radnor, PA, USA catalog no. BDH1133) was added and gently triturated to dissolve the MTT crystals. Absorbance of all wells was read at 570 nm by a SpectraMax M5e Microplate Reader (Molecular Devices, San Jose, CA, USA).

### 4.6. Viability Assay

NB cell lines were plated and allowed to adhere for 24 h prior to treatment with M344, vorinostat, or vehicle control for 24, 48, or 72 h. Dead cells were stained in an aliquot of each treatment group with trypan blue, 0.4% (Thermo Fisher Scientific, catalog no. 15250-061) and counted on a hemocytometer. Percentages of viable cells were calculated as (unstained cells/total counted cells) × 100.

### 4.7. Transwell Migration

NB cell lines were resuspended at 100,000 cells in 200 μL complete medium lacking FBS (serum-free medium) and containing M344, vorinostat, or vehicle control in 8 μm Transwell inserts (Corning Incorporated, Corning, NY, USA catalog no. 353097). Transwell inserts were placed in a 24-well plate containing 2 mL/well complete medium with 20% FBS as a migration stimulus. Cells were allowed to migrate for 24 h prior to inserting staining with a Hema 3 Stat Pack Wright–Giemsa staining kit (Thermo Fisher Scientific, catalog no. 122-911). Stained inserts were mounted on slides and visualized with a Nikon Eclipse E400 microscope (Nikon Instruments, Melville, NY, USA) at 200× magnification, and 3 random fields were selected for counting.

### 4.8. Bone Marrow Isolation and Macrophage Selection

Fibulas and tibias excised from euthanized C57BL/6 (The Jackson Laboratory, Bar Harbor, ME, USA strain no. 000664) mice had one epiphysis from each bone removed using sharp scissors. With the open marrow cavity facing downward, the bones were placed into 0.5 mL microcentrifuge tubes that had been previously punctured at the bottom with a 20G needle. The tubes were sealed, then nested inside 1.5 mL microcentrifuge tubes containing 150 μL PBS (Thermo Fisher Scientific, catalog no. 70011-044). The nested tubes were centrifuged at 10,000 RPM for 1 min at 4 °C in an Eppendorf 5417R centrifuge (Eppendorf, Hamburg, Germany). The bone marrow was then washed with 1 mL of cold PBS and transferred to a 15 mL conical tube, followed by the addition of 10 mL PBS. The suspension was centrifuged at 1200 RPM for 5 min at 4 °C in an Eppendorf 5810R centrifuge. Within a laminar flow cell culture hood, the PBS wash was aspirated, and red blood cells were lysed by addition of 900 μL of hypotonic water (Thermo Fisher Scientific, catalog no. 10977-015) and gentle mixing. Immediately following, 100 μL 10× PBS was slowly incorporated to create an isotonic solution. The cell suspension was filtered through a 70 μm cell strainer (Thermo Fisher Scientific, catalog no. 22363548) into a 50 mL conical tube and centrifuged again at 1200 RPM for 5 min at 4 °C. After removing the supernatant, the cell pellet was resuspended in RPMI 1640 complete medium supplemented with 10% L-929-conditioned media to generate complete macrophage medium. Bone marrow contents were cultured in this medium for 7 days to promote monocyte differentiation into macrophages.

### 4.9. Flow Cytometry

For flow cytometry assays of NB cells, the cells were dissociated from tissue culture flasks with TrypLE™ Express Enzyme (Thermo Fisher Scientific, catalog no. 12604-013). After detachment, the cells were resuspended in complete medium and pelleted by centrifugation at 1500 RPM for 5 min in a 4 °C cooled Eppendorf 5810R centrifuge. Following centrifugation, the cell pellet was resuspended in PBS (Thermo Fisher Scientific, catalog no. 70011-044) at a concentration of 1 × 10^6^ cells/100 µL, and 100 µL aliquots were distributed into the wells of a 96-well plate. The plate was centrifuged again at 1500 RPM for 5 min, and the supernatant was discarded. For NB MHC I staining, cells were incubated in PBS containing LIVE/DEAD Fixable Blue Dead Cell Stain (Thermo Fisher Scientific, catalog no. L34962) and the pan-MHC I antibody W6/32 (donated by Dr. Ted Hansen, Washington University, St. Louis, MO, USA) for 30 min at 4 °C. Following removal of the supernatant, the cells were resuspended in PBS and centrifuged at 1500 RPM for 5 min in a 4 °C centrifuge as 1 wash cycle. Cells were washed a total of 3 times prior to secondary antibody incubation, following the same procedure used in primary antibody staining and 3 subsequent wash cycles. Cells were fixed in 1% paraformaldehyde (PFA, MilliporeSigma, catalog no. P6148). Apoptotic cells were stained with CellEvent™ Caspase-3/7 Green Flow Cytometry Assay Kit (Thermo Fisher Scientific, catalog no. C10427), following the manufacturer’s protocol.

Murine macrophages were detached with TrypLE™ Express Enzyme (Thermo Fisher Scientific, catalog no. 12604-013) and resuspended in complete macrophage medium. The cell concentration was adjusted to 2 × 10^6^ cells/100 µL for staining. Cell-surface staining for selection of live, mature macrophages was performed using PBS with CD11b and F4/80 antibodies (Miltenyi Biotec, catalog no. 130-113-243 and 130-113-243, respectively) and LIVE/DEAD Fixable Blue Dead Cell Stain. Macrophages underwent 3 washes prior to fixation and permeabilization for intracellular staining. Cells were fixed in 2% PFA for 15 min at 4 °C, followed by incubation in permeabilization buffer (0.5% Tween 20 [Thermo Fisher Scientific] in PBS) for 15 min at room temperature. The inflammatory status of macrophages was determined by incubation for 30 min at 4 °C, with antibodies for intracellular pro-inflammatory macrophage marker iNOS (Thermo Fisher Scientific, catalog no. 50-112-2349) and anti-inflammatory macrophage marker Arg1 (Thermo Fisher Scientific, catalog no. 17-369-782) diluted in permeabilization buffer. Cells were subjected to 3 wash cycles followed by flow cytometric analysis at the University of Nebraska Medical Center Flow Cytometry Core with a BD LSR II Flow Cytometer (BD Biosciences, Franklin Lakes, NJ, USA). Resulting data were analyzed with FlowJo^TM^ version 10.10.0 (BD Biosciences).

### 4.10. Cell Cycle Analysis

M344- and vehicle-treated NB cells were detached from culture plates using Trypsin-EDTA (Thermo Fisher Scientific, catalog no. 25300-054), resuspended in complete medium, washed with 1 cycle of centrifugation at 2000 RPM for 5 min in an Eppendorf 5810R centrifuge, and resuspended in 1 mL PBS (Thermo Fisher Scientific, catalog no. 70011-044). After an additional round of centrifugation, cells were resuspended in 200 μL PBS and fixed by the slow addition of 800 μL ethanol (Decon Laboratories, King of Prussia, PA, USA catalog no. 2701) with constant agitation from a vortex mixer. Cells were incubated at 4 °C for 1 h prior to centrifugation at 2400 RPM for 4 min. Following removal of the supernatant, cells were washed once in PBS and spun at 2400 RPM for 4 min. Cells were resuspended in 100 μL pH 7.6 citrate buffer (0.250 M sucrose [Thermo Fisher Scientific, catalog no. BP-2201], 40 mM sodium citrate tribasic dihydrate [MilliporeSigma, catalog no. C3434], 5% *v*/*v* DMSO [Thermo Fisher Scientific, D-128-1]), and incubated at room temperature for 10 min. For permeabilization, 300 μL pH 7.6 NP-40 and trypsin solution (3.4 mM sodium citrate tribasic dihydrate, 0.1% *v*/*v* NP-40 [MilliporeSigma, catalog no. CA630], 1.5 mM spermine [MilliporeSigma, catalog no. 5-2876], 0.5 mM Tris [Thermo Fisher Scientific, catalog no. BP15201], and 0.003% *w*/*v* trypsin [MilliporeSigma, catalog no. T0134]) was added prior to vortexing and an additional 10 min incubation at room temperature. For RNA degradation and trypsin inactivation, 300 μL of ribonuclease A and trypsin inhibitor solution (0.05% *w*/*v* trypsin inhibitor [MilliporeSigna, catalog no. T9253], 0.01% *w*/*v* ribonuclease A [MilliporeSigma, catalog no. R4875], 3.4 mM sodium citrate tribasic dihydrate, and 0.1% *v*/*v* NP-40, 1.5 mM spermine, 0.5 mM Tris) were added prior to a 10 min incubation at room temperature. Nucleic acid content was stained with propidium iodide solution (3.4 mM sodium citrate tribasic dihydrate [MilliporeSigma, catalog no. C3434], 0.1% *v*/*v* NP-40, 1.5 mM + 0.01% *w*/*v* spermine, 0.5 mM Tris, and propidium iodide [MilliporeSigma, catalog no. P-4170]), followed by filtration through a 35 μm cell-strainer capped 5 mL round-bottom tube (Corning Incorporated, catalog no. 352235). In the University of Nebraska Medical Center Flow Cytometry Core, the stained cells were analyzed utilizing a BD LSR II Flow Cytometer (BD Biosciences), and ensuing data were evaluated with FlowJo^TM^ version 10.10.0 (BD Biosciences).

### 4.11. In Vivo NB Model

All handling, housing, and experimental treatment of mice were conducted within the standards of the University of Nebraska Medical Center Institutional Animal Care and Use Committee (IACUC) protocol #18-011-02-EP, and reporting of resulting data from these mouse studies adheres to the ARRIVE 2.0 guidelines for reporting of in vivo experiments [65]. Mice were housed in the same room within a Biosafety Level 2 (BSL-2) animal facility at the University of Nebraska Medical Center with rodent bedding enrichment and ad libitum food and water. For a murine model of NB, female A/J mice (The Jackson Laboratory, strain no. 000646), 4 weeks of age, were purchased and then acclimated for 2 weeks prior to subcutaneous injection with 1 × 10^6^ low-passage Neuro-2a cells in 100 μL PBS (Thermo Fisher Scientific, catalog no. 10-010-031) into the shaved left flank. With a target of 10 mice per group, as calculated by power analysis with significance level (alpha) of 0.05 and 80% power in assessing difference in tumor volumes, 120 mice were injected for these studies. Tumors were measured with digital calipers (Avantor, catalog no. 36934-152) in two dimensions: length (rostral to caudal) and width (medial to lateral). Tumor volume was calculated with the following formula: volume = [(width^2^) × length]/2. Mice were euthanized if either tumor dimension (length or width) reached greater than 2 cm, the total tumor volume became greater than 1 cm^3^, or body weight loss exceeded 20% of starting weight. Mouse tumor volumes were recorded 3 days per week and body weight 5 days per week. Once mice developed palpable tumors and could be randomly divided into treatment groups with approximately equal average tumor volumes by a researcher (G.L.B.), treatments were assigned to each group by a different researcher (K.R.D.), who was independent from (and blinded to) the subsequent outcome assessments and data analysis. Mice that did not develop palpable tumors or developed a tumor with volume <10 mm^3^ or >60 mm^3^ at group allocation were excluded from these studies. The experimental unit (n) for each treatment group is reported as a single animal. Treatments were then prepared and immediately delivered by a researcher (G.L.B.) in 100 μL intraperitoneal injection with solo therapy at 50 mg/kg M344 3 times per week, 10 mg/kg M344 5 times per week, 10 mg/kg topotecan 3 times over 9 days, 40 mg/kg cyclophosphamide 1 time per week, or vehicle control 5 times per week with equivalent DMSO volume to M344 group. Dual therapy groups received either 10 mg/kg M344 5 times per week with 10 mg/kg topotecan 3 times over 9 days or 10 mg/kg M344 5 times per week with 40 mg/kg cyclophosphamide 1 time per week. Researcher G.L.B. conducted the outcome assessment and data analysis by approaches which were reviewed and confirmed by the corresponding author (J.C.S.).

### 4.12. Statistical Analysis

GraphPad Prism version 10.2.3 (GraphPad Software Inc., La Jolla, CA, USA) was utilized for conducting statistical testing and creating graphical visuals. To compare means between two or more independent groups, an independent t-test or one-way ANOVA with post hoc analysis was utilized as indicated. Significance was determined by *p* value < 0.05, and *p* values are represented in graphics as * *p* < 0.05, ** *p* < 0.01, *** *p* < 0.001, and **** *p* < 0.0001. Sample size (n) for in vitro studies is expressed as number of biological replicates.

## 5. Conclusions

In summary, our study highlights the HDAC inhibitor M344 as a novel and efficacious potential treatment modality for the pediatric cancer NB, particularly addressing the need for treatments that combine efficacy with reduced toxicity and long-lasting effects. We demonstrated that M344 exhibits significant cytotoxic and cytostatic activity, suppresses NB cell migration, and positively modulates NB and immune cells for enhanced elimination of NB tumors. These findings underscore the potential not only to alleviate tumor burden but also to prevent metastasis and alleviate immune suppression without immune cell toxicity. Additionally, M344 displayed an ability in vivo to improve tolerability of standard chemotherapy and mitigate tumor rebound, supporting its role as a complementary agent. These findings position M344 as a promising therapeutic option, and in total our work supports further investigation of M344 as a component of NB therapy.

## Data Availability

The data produced and/or analyzed in this study can be obtained from the corresponding author upon reasonable request.

## Abbreviations

The following abbreviations are used in this manuscript:

Arg1	arginase-1
BSL-2	Biosafety Level 2
COG	Children’s Oncology Group
DMSO	dimethyl sulfoxide
FBS	fetal bovine serum
FDA	Food and Drug Administration
FPKM	Fragments Per Kilobase of transcripts per Million
GAPDH	glyceraldehyde-3-phosphage dehydrogenase
GEO	Gene Expression Omnibus
HDAC	histone deacetylase
IACUC	Institutional Animal Care and Use Committee
iNOS	inducible nitric oxide synthase
INRGSS	International Neuroblastoma Risk Group Staging System
INSS	International Neuroblastoma Staging System
M344	4-(dimethylamino)-N-(7-(hydroxyamino)-7-oxoheptyl)benzamide
MFI	median fluorescence intensity
MHC I	major histocompatibility complex class I
MTT	3-(4,5-dimethylthiazol-2-yl)-2,5-diphenyltetrazolium bromide
NB	neuroblastoma
PFA	paraformaldehyde
RPM	revolutions per minute
SEM	standard error of the mean
SIRT	sirtuin
